# The Deletion of *rnhB* in *Mycobacterium smegmatis* Does Not Affect the Level of RNase HII Substrates or Influence Genome Stability

**DOI:** 10.1371/journal.pone.0115521

**Published:** 2015-01-20

**Authors:** Alina E. Minias, Anna M. Brzostek, Piotr Minias, Jaroslaw Dziadek

**Affiliations:** 1 Department of Genetics and Physiology of *Mycobacterium*, Institute of Medical Biology, Polish Academy of Sciences, Lodz, Poland; 2 Department of Microbiology, Biotechnology and Immunology, University of Lodz, Lodz, Poland; 3 Department of Teacher Training and Biodiversity Studies, University of Lodz, Lodz, Poland; Keio University, JAPAN

## Abstract

RNase HII removes RNA from RNA/DNA hybrids, such as single ribonucleotides and RNA primers generated during DNA synthesis. Both, RNase HII substrates and RNase HII deficiency have been associated with genome instability in several organisms, and genome instability is a major force leading to the acquisition of drug resistance in bacteria. Understanding the mechanisms that underlie this phenomenon is one of the challenges in identifying efficient methods to combat bacterial pathogens. The aim of the present study was set to investigate the role of *rnhB*, presumably encoding RNase HII, in maintaining genome stability in the *M. tuberculosis* model organism *Mycobacterium smegmatis*. We performed gene replacement through homologous recombination to obtain mutant strains of *Mycobacterium smegmatis* lacking the *rnhB* gene. The mutants did not present an altered phenotype, according to the growth rate in liquid culture or susceptibility to hydroxyurea, and did not show an increase in the spontaneous mutation rate, determined using the Luria-Delbrück fluctuation test for streptomycin resistance in bacteria. The mutants also did not present an increase in the level of RNase HII substrates, measured as the level of alkaline degradation of chromosomal DNA or determined through immunodetection. We conclude that proteins other than RnhB proteins efficiently remove RNase HII substrates in *M. smegmatis*. These results highlight differences in the basic biology between *Mycobacteria* and eukaryotes and between different species of bacteria.

## Introduction

RNase H proteins are ubiquitous enzymes present is eukaryotes, prokaryotes, archeons and viruses. These proteins are involved in the removal of RNA from RNA/DNA hybrids present during several cellular processes, including DNA replication, repair and transcription. Based on differences in the amino acid sequence, prokaryotic RNase H proteins have been divided into three classes and two types. RNase HI represents RNase H type 1 enzymes, whereas RNases HII and HIII represent RNase H type 2 enzymes. RNase HI and RNase HII are present in various organisms, while RNase HIII has only been detected in a limited number of bacteria, including *Bacillus subtilis* [1] and *Chlamydophila pneumoniae* [2]. The genome of *Mycobacterium smegmatis* encodes one gene with RNase HII fold- *rnhB*, similarly as the genome of *Mycobacterium tuberculosis* [3].

The results of *in vitro* experiments suggested that RNase HII recognize the RNA to DNA transition [4, 5]. Currently, two types of substrates for prokaryotic RNase HII have been identified. The first type of RNase HII substrates are RNA primers generated during DNA replication. Although the major pathway for Okazaki primer removal in eukaryotes is independent of RNase H activity [6], these structures might also be removed through RNase HII. Using an *in vitro* model, it was demonstrated that RNase H type 2 proteins cleave RNA primers in Okazaki fragments, leaving the last ribonucleotide of the primer attached to the DNA [4, 7, 8, 9], which is subsequently cleaved by FEN1 [10]. This mechanism was consistent with the observation that human RNase H2 localizes to replication foci [11]. The involvement of RNase H type 2 in Okazaki fragment processing has also been confirmed in archaea [12, 13]. However, in *Escherichia coli*, the enzyme responsible for the removal of RNA primers during Okazaki fragment maturation is polymerase I (PolI), encoded by *polA* [14, 15, 16]. Apart from PolI, RNase H proteins have also been implicated in RNA primer removal in bacteria [17].

The second type of substrate for RNase HII are single ribonucleotides embedded within DNA duplex. Until recently, the incorporation of ribonucleotides other than Okazaki fragments within the DNA double helix has been a neglected biological phenomenon. However, several recent studies have shown that ribonucleotides are indeed present within DNA, and ribonucleotide triphosphates incorporation is evolutionarily conserved from bacteria to eukaryotes. In fact, ribonucleotides might be the most common lesion in the DNA double helix. For example, the replicative polymerases α, δ, and ε in *S. cerevisiae* have been shown to insert one ribonucleotide for every 625, 5000 and 1250 deoxyribonucleotide triphosphates, respectively. This activity results in the incorporation of approximately 10 thousand ribonucleotides per round of replication [18].

In yeast, the removal of ribonucleotides embedded within the DNA double helix primarily occurs through RNase H2 [18, 19, 20] and to some extent, RNase H1 [19] and topoisomerase I [21]. Although genetic analyses in yeast have excluded the involvement of nucleotide excision repair, the minor involvement of base excision repair could not be ruled out [19]. Intriguingly, contrasting observations have been made in *E. coli* where, apart from RNase HII activity, the removal of ribonucleotide monophosphate primarily involved nucleotide excision repair, while the involvement of base excision repair and mismatch repair (MMR) was minimal [22].

Both, increased RNase HII substrates and RNase HII deficiency have been associated with genome instability in several organisms. Thus, the aim of the present study was to investigate whether *rnhB*, presumably encoding RNase HII, influences RNase HII substrate levels and the genome stability in the *M. tuberculosis* model organism *M. smegmatis*. *M. smegmatis* genome contains two putative RNases H- one RNase H class I enzyme encoded by *rnhA* gene one RNase H class II enzyme encoded by *rnhB* gene. Additionally, a two domain protein encoded by MSMEG4309 is homologous to Rv2228c found in *M. tuberculosis*, which encodes an RNase HI domain and a domain involved in cobalamin biosynthesis [23]. The protein expressed from MSMEG5849 has been shown to present RNase HII activity as well as being capable of pGpp synthesis [24]. Therefore, the genome of *M. smegmatis* might encode two RNases H class I and two RNases H class II.

Genome instability is a major force leading to the acquisition of drug resistance in bacteria. Understanding the mechanisms that underlie this instability is a challenge in identifying efficient methods to combat bacterial pathogens. We did not observe an increase in the levels of RNase HII substrates or mutation rates in mutant strains deficient in *rnhB*. Hence, we suspect that proteins other than RnhB proteins are involved in the removal of RNase HII substrates in *M. smegmatis*.

## Materials and Methods

### *In silico* analysis

The sequences for the genes presumably encoding RNase H proteins in *M. smegmatis*, *E. coli* and *M. tuberculosis* were identified and obtained from the PubMed database. The span of the domains was defined using the Simple Modular Architecture Research Tool (SMART). The homology between the domains was estimated using the National Center for Biotechnology Information (NCBI) BLAST tool. The alignments were visualized using Clustal Omega and ESPript 2.2.

### Bacterial culture

*M. smegmatis* cultures were grown in nutrient broth (NB; Difco) during gene replacement or in 7H9 broth (Becton, Dickinson and Company) supplemented with OADC (Becton-Dickinson) and 0.05% Tween 80 (Sigma) for the determination of growth rates. Where necessary, the medium was supplemented with antibiotics and/or other supplements at the following concentrations: 25 µg/ml kanamycin (Sigma), 40 µg/ml X-gal (Sigma), 400 µg/ml hydroxyurea (HU) (Sigma), 0.4% succinate (Sigma), 12.5 µg/ml streptomycin (Serva), and 2% sucrose (Sigma). The *M. smegmatis* strains used in this study are listed in Table 1.

**Table 1:** List of the *M. smegmatis* strains used in this study.

**Strain**	**Characteristics**
*M. smegmatis* mc^2^ 155	Reference strain
∆*rnhB*	*rnhB* deletion mutant of mc^2^ 155
∆*rnhA*/∆4305/∆*rnhBattB::rnhA*	Derivative of mc^2^ 155 carrying deletions in the *rnhA*, MSMEG4305 and *rnhB* genes complemented with the full-length *rnhA* gene under the natural promoter at the *attB* site, Hyg^R^
∆*rnhA*/∆4305/∆*rnhBattB*::4305	Derivative of mc^2^ 155 carrying deletions in the *rnhA*, MSMEG4305 and *rnhB* genes complemented with full-length MSMEG4305 gene under the acetamide promoter at the *attB* site, Hyg^R^

To determine the growth rates, bacterial cells were transferred to fresh NB medium and incubated until the cultures reached an OD_600_ between 0.6 and 0.9. Aliquots of these seed cultures were inoculated into fresh 7H9 broth supplemented with OADC or 7H9 broth supplemented with OADC and HU at starting OD_600_ = 0.05. The cultures were incubated at 37°C with vigorous shaking for 24–48 hours. At desired time intervals, samples of the cultures were harvested and analyzed using a spectrophotometer (Pharmacia Biotech Ultrospec 2000). To assess the number of colony forming units, the samples were serially diluted into fresh NB broth and plated onto non-selective NB medium. The cultures were incubated at 37°C until obtaining visible colonies. Each experiment was performed at least in triplicate. To determine the cell length, samples of the cultures were harvested at 24 h and placed onto glass slides, followed by heat fixing and analysis using a Nikon Eclipse TE2000 microscope.

For susceptibility testing, cultures at starting OD_600_ = 0.05 were incubated at 37°C with vigorous shaking until reaching the logarithmic phase of growth (OD_600_ = 0.7–0.8) and the stationary phase of growth (24 hours). The logarithmic and stationary phase cultures were serially diluted, and 5 µl of each dilution was plated onto 7H10 medium supplemented OADC, succinate and 6, 10.5, 15 and 20 mM HU. As a control, 5 µl of each dilution was plated onto 7H10 medium without the addition of HU.

### *M. smegmatis* mutants

The mutants were obtained using a gene replacement protocol as previously described [25, 26, 27]. The procedure required the construction of gene replacement and complementation plasmids. The primers used for the generation of the mutants are listed in Table 2.

**Table 2:** Primers used in this study.

**Primers used to generate the mutants**				
**Name**	**Sequence**	**Primer pair**	**Introduced restriction sites**	**Destination**
MsGR1rnhA	TCGAGGGCAAGCTGCGCGAC	MsGR2rnhA	-	Gene replacement *rnhA*
MsGR2rnhA	CGTAGCACCGCACCCCAGCC	MsGR1rnhA	-	Gene replacement *rnhA*
MsGR3rnhA	CGGGATCCGTGCGCGCGCCACCAGGTC	MsGR4rnhA	BamHI	Gene replacement *rnhA*
MsGR4rnhA	GAAGCTTCCGCGAGGGGCCGAACACC	MsGR3rnhA	HindIII	Gene replacement *rnhA*
GR1MsRnhAII-KpnI	GGTACCCCGCCGACGATGATGCTGTC	GR2MsRnhAII-BamH	KpnI	Gene replacement MSMEG4305
GR2MsRnhAII-BamH	CAAGCGGCGCAACGGGATC	GR1MsRnhAII-KpnI	-	Gene replacement MSMEG4305
GR3MsRnhAII-BamH	CGGGATCCTACACAACCGCGCCGTAGCC	GR4MsRnhAII-Hind	BamHI	Gene replacement MSMEG4305
GR4MsRnhAII-Hind	GCAAGCTTTCGCTGCTGGGTGCCGTGAC	GR3MsRnhAII-BamH	HindIII	Gene replacement MSMEG4305
MsGR1rnhB	AACTGCAGACTACCTGCGCGAGCTCCGTG	MsGR2rnhB	PstI	Gene replacement *rnhB*
MsGR2rnhB	GAAGCTTCGCAGACCCGACGATTTCCG	MsGR1rnhB	HindIII	Gene replacement *rnhB*
RnHBgr3a	GCGAATTCCGTCGCTTCCCGTGATCGGC	RnHBgr4a	HindIII	Gene replacement *rnhB*
RnHBgr4a	GGGGTACCAAGGGTTTTGCCGCATCCGC	RnHBgr3a	KpnI	Gene replacement *rnhB*
MsA1–5562-BglIIs	CAGATCTGTGAACCACCGGCACCACGCC	MsA1–5562-XbaI	BglII	Complementation *rnhA*
MsA1–5562-XbaI	CTCTAGATGGTGGTCGGCCTGGCGGG	MsA1–5562-BglIIs	XbaI	Complementation *rnhA*
MsrnhAIIPace-sBglII	CAGATCTGTGAAGGTTCTCGTCGAGGCCGAC	MsrnhAII-rev-Xba	BglII	Complementation MSMEG4305
MsrnhAII-rev-Xba	CTCTAGATGCACTCGTGAGCTACAGGTACGC	MsrnhAIIPace-sBglII	XbaI	Complementation MSMEG4305

**Primers used for obtainment of probes for Southern blotting**				
**Gene name**	**Primer name**	**Sequence**	**Primer pair**	**Restriction enzyme**
*rnhA* before complementation	coDCOH1s	GTGAACCACCGGCACCACGCC	coDCOH1r	PvuI
*rnhA* before complementation	coDCOH1r	GGCGGGCAACAAGCTCAACGG	coDCOH1s	PvuI
*rnhA* after complementation	MsGR3rnhA	CGGGATCGGTGCGCGCGCCACCAGGTC	MsRnhA1probe-r-delikom	BamHI, PvuI, XbaI
*rnhA* after complementation	MsRnhA1probe-r-delikom	TGTGGGCCGGTGCGGTGG	MsGR3rnhA	BamHI, PvuI, XbaI
MSMEG4305 before complementation	MsrnhAII Pace-sBglII	CAGATCTGTGGAAGGTTCTCGTCGAGGCCGAC	MsrnhAIIrev-Xba	BamHI, PvuII

Briefly, for gene replacement, the sequences flanking the desired deletion were amplified through PCR and consecutively introduced into p2NIL plasmid, followed by the introduction of the PacI suicidal cassette excised from vector pGOAL17. For complementation under an acetamide promoter, a native copy of the gene of interest was amplified through PCR and introduced into pJAM2. The gene and the acetamide promoter were subsequently excised from pJAM2 and introduced into pMV306Hyg. All cloning was performed in *E. coli* Top10 cells. The plasmids were introduced into mycobacterial cells, which were further subjected to a multistep selection process for the detection of the mutants.

### Southern blotting

The primers used to obtain the probes and the restriction enzymes used for the digestion of the genomic DNA are listed in Table 2.

### Luria-Delbrück fluctuation test

The Luria-Delbrück fluctuation test was performed as previously described [28]. Briefly, 100 ml of 7H9 medium supplemented with OADC and succinate was inoculated with approximately 100 bacteria per ml, based on the determination of colony forming units. To avoid clumping, the inoculum was passed through a syringe needle. The inoculated medium was immediately divided into 30 cultures of 3 ml each. After 72 hours at 37°C with vigorous shaking, the contents of each tube were centrifuged. A total of 26 of cultures were plated onto plates supplemented with streptomycin, while the remaining four cultures were serially diluted and used to assess the number of colony forming units. The plates were incubated for 72 h at 37°C, and subsequently the number of colony forming units on each plate was counted. The results were analyzed using the FALCOR (Fluctuation Analysis Calculator) software program.

### Alkaline degradation of chromosomal DNA

The bacterial cells were transferred to fresh NB medium and cultured until reaching an OD_600_ between 0.6 and 0.9. Aliquots of these seed cultures were inoculated into fresh 7H9 broth supplemented with OADC at a starting OD_600_ = 0.05. The cultures were incubated at 37°C with vigorous shaking for 24 hours. Subsequently, the cells were harvested through centrifugation for 10 min at 8000 x g at 4°C and subjected to DNA isolation. The DNA solution was incubated in 0.3 M NaOH (Sigma) for 2 h at 55°C. As a control, the DNA solution was incubated in 0.3 M NaCl for 2 h at 55°C. Subsequently, the samples were resolved on 1% alkaline agarose gels. The level of DNA fragmentation was visually assessed through a comparison of the mobility of the DNA fragments on the gel in relative reference to the size standard (GeneRuler 1-kb DNA ladder, Thermo Scientific).

### Immunodetection of RNA/DNA hybrids

The bacteria were grown in liquid culture for 24 hours in 7H9 medium supplemented with OADC and succinate. Subsequently, 3 ml of each culture was centrifuged and subjected to total nucleic acid extraction. A 50-µl sample of the bacterial cells was resuspended in 200 µl of TE buffer (10 mM Tris-HCl (Serva), pH 8.0 and 1 mM EDTA (Serva)). Approximately 50 µl of zirconia beads (BioSpec Products) was added, and the samples were homogenized using an MP Fast Prep homogenizer. Subsequently, an equal volume of phenol:chloroform (Sigma) was added, and after vigorous shaking, the samples were centrifuged at 14000 x g for 10 min. The aqueous phase was transferred to a new Eppendorf tube, and three volumes of ethanol (Sigma) were added. The samples were incubated at -20°C overnight and centrifuged for 10 min at 14000 x g. The pellet was dried at room temperature for approximately 15 min and resuspended in 100 µl of water. The samples were serially diluted, and 2 µl of each dilution was placed onto an Amersham Hybond N+ nylon membrane (GE Healthcare Life Sciences). The membrane was air-dried and blocked with 5% milk in PBST (137 mM NaCl (Sigma); 2.7 mM KCl (Sigma); 10 mM Na_2_HPO_4_ • 2 H_2_O (Sigma); 2 M KH_2_PO_4_ (Sigma) pH 7.4; and 0.02% Tween 20 (Sigma)) at room temperature. The membrane was washed three times in PBST for 5 min at room temperature and incubated overnight at 4°C with a primary monoclonal mouse antibody against the RNA/DNA hybrid (D5H6) (Covalab) suspended in PBST containing 1% bovine serum albumin. Subsequently, the membrane was washed three times in PBST at room temperature and incubated with a secondary anti-mouse rabbit IgG antibody (Sigma) conjugated with peroxidase suspended in PBST containing 1% milk at room temperature. The membrane was washed three times in PBST and covered with ECL detection reagent (Amersham Biosciences). The excess reagent was removed, and the membrane was tightly wrapped in Saran wrap and exposed to X-ray film (Thermo Scientific) for 1 minute. The film was developed using a Kodak Medical X-ray Processor.

### Statistical analysis

To determine the growth rates of the analyzed strains, logistic or quadratic curves were fitted to the optical density measurements obtained from the liquid cultures. When the growth rates were determined after 48 h, we fitted logistic curves of the form OD = A/[1 + B*exp(-KT)], where OD is the optical density at time T, A is an asymptotic value, B is a constant of integration, and K is the growth rate constant. When the growth rates were estimated after 24 h, we fitted quadratic curves of the form OD = K*T^2^ + OD_0_, where OD is the optical density at time T, OD_0_ is the starting optical density, and K is the growth rate constant. Parameter K from the fitted curves was used as an indicator of the growth rate for each strain. A t-test was used to assess differences in the growth rates between the strains. The non-parametric Kruskal-Wallis test was used to compare the mutation rates between the strains. All statistical analyses were performed using Statistica 10.0 software (StatSoft, Tulsa, OK, USA).

## Results

### The *M. smegmatis* genome of encodes a protein with an RNase HII fold

**Figure 1:**
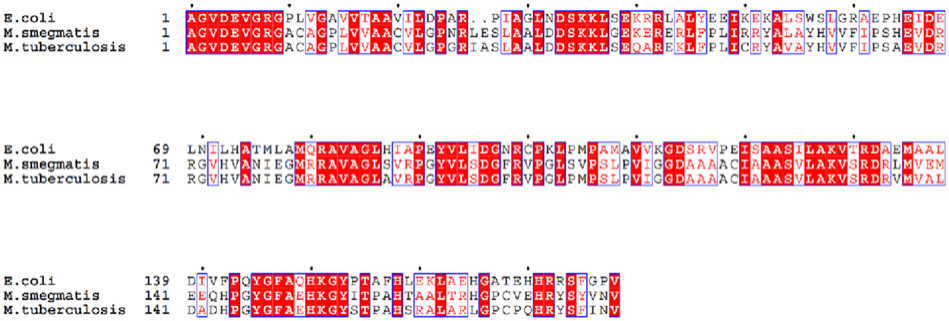
Sequence comparisons of the RNase HII domains in the RnhB proteins of *E. coli* K12_MG1655, *M. smegmatis* mc^2^ 155 and *M. tuberculosis* H37Rv.

Based on the genome sequence of *M. smegmatis* mc^2^ 155 deposited in the NCBI database, we identified an *rnhB* gene, presumably encoding RNase HII. The RNase HII domain of *M. smegmatis* mc^2^ 155 RnhB shares 61% sequence homology with the same domain of *E. coli* K12_MG1655 RnhB with 98% of query cover of *M. smegmatis* RnhB (Fig. 1A). RnhB has also been identified in the genome of *M. tuberculosis* H37Rv. The RNase HII domains of these species share 91% amino acid sequence homology with 100% query cover of *M. smegmatis* RnhB (Fig. 1B).

### Generating *rnhB* deficient mutants

**Figure 2:**
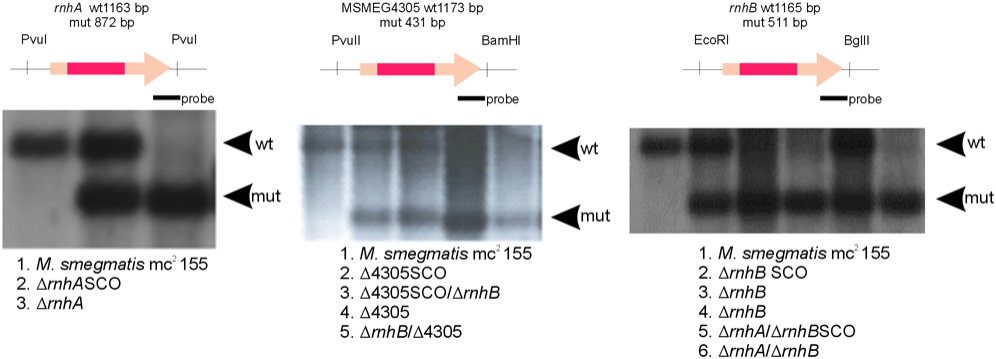
Southern blot analyses confirming the deletions in the single and double RNase H mutants of *M. smegmatis*. We used the gene replacement through homologous recombination technique to obtain single and double mutants deficient in *rnhB* and/or either *rnhA* or MSMEG4305. The ∆*rnhA*/∆*rnhB* mutant was obtained through the introduction of the *rnhB* gene replacement plasmid into the ∆*rnhA*-deficient strain. The mutant ∆4305/∆*rnhB* was obtained through the introduction of the MSMEG4305 gene replacement plasmid into the ∆*rnhB* strain. The intermediate steps of the gene replacement procedure are denoted SCO.

**Figure 3:**
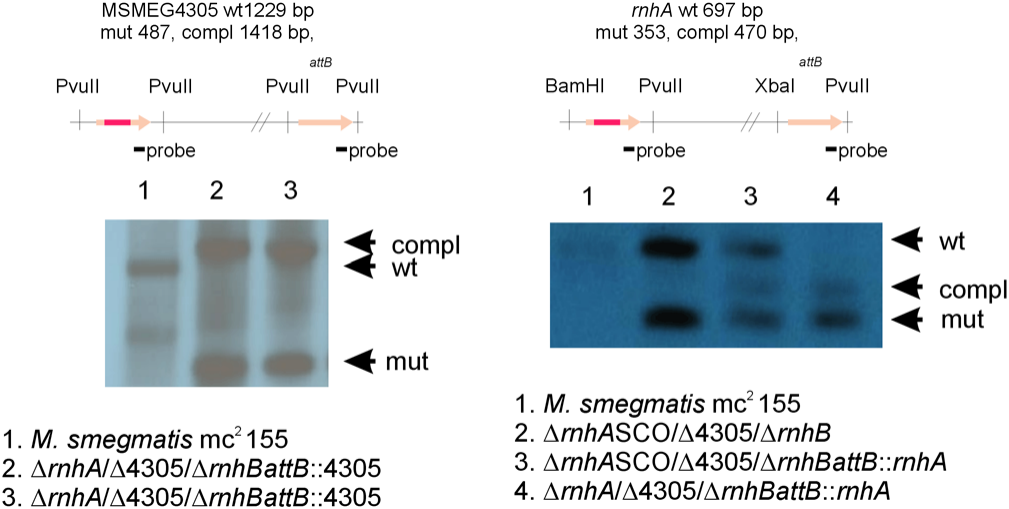
Southern blot analyses confirming the generation of ∆rnhA/∆4305/∆rnhBattB::rnhA and ∆rnhA/∆4305/∆rnhBattB::4305 strains. We used the double mutant strains ∆rnhA/∆rnhB and ∆4305/∆rnhB to obtain the triple conditional mutants ∆rnhA/∆4305/∆rnhBattB::rnhA and ∆rnhA/∆4305/∆rnhBattB::4305. The intermediate steps of the gene replacement procedure are denoted SCO.

We used gene replacement through homologous recombination to obtain genetic mutants with large deletions within the sequence of the predicted RNase HII gene, *rnhB*. We also obtained mutants deficient not only in the *rnhB* gene, but also in the genes encoding predicted RNase HI proteins (*rnhA* and MSMEG4305). The *rnhB*-deficient mutants were confirmed through Southern blot analysis (Fig. 2 and Fig. 3). We obtained a single ∆*rnhB* mutant. The two double mutants, ∆*rnhA*/∆*rnhB* and ∆4305/∆*rnhB*, were used to generate the triple conditional mutants ∆*rnhA*/∆4305/∆*rnhBattB*::*rnhA* and ∆*rnhA*/∆4305/∆*rnhBattB*::4305, where the expression of *rnhA* or MSMEG4305, controlled through the acetamide promoter, is inhibited in the presence of succinate. Because even succinate inhibition did not inhibit complemented gene expression below the native level (data not shown), the data obtained from the analysis of these strains only reflects the consequences of *rnhB* deletion and not consequences of the deletion of predicted RNase HI-encoding genes.

### Phenotypic analysis of the *M. smegmatis* mutants lacking *rnhB*

**Figure 4:**
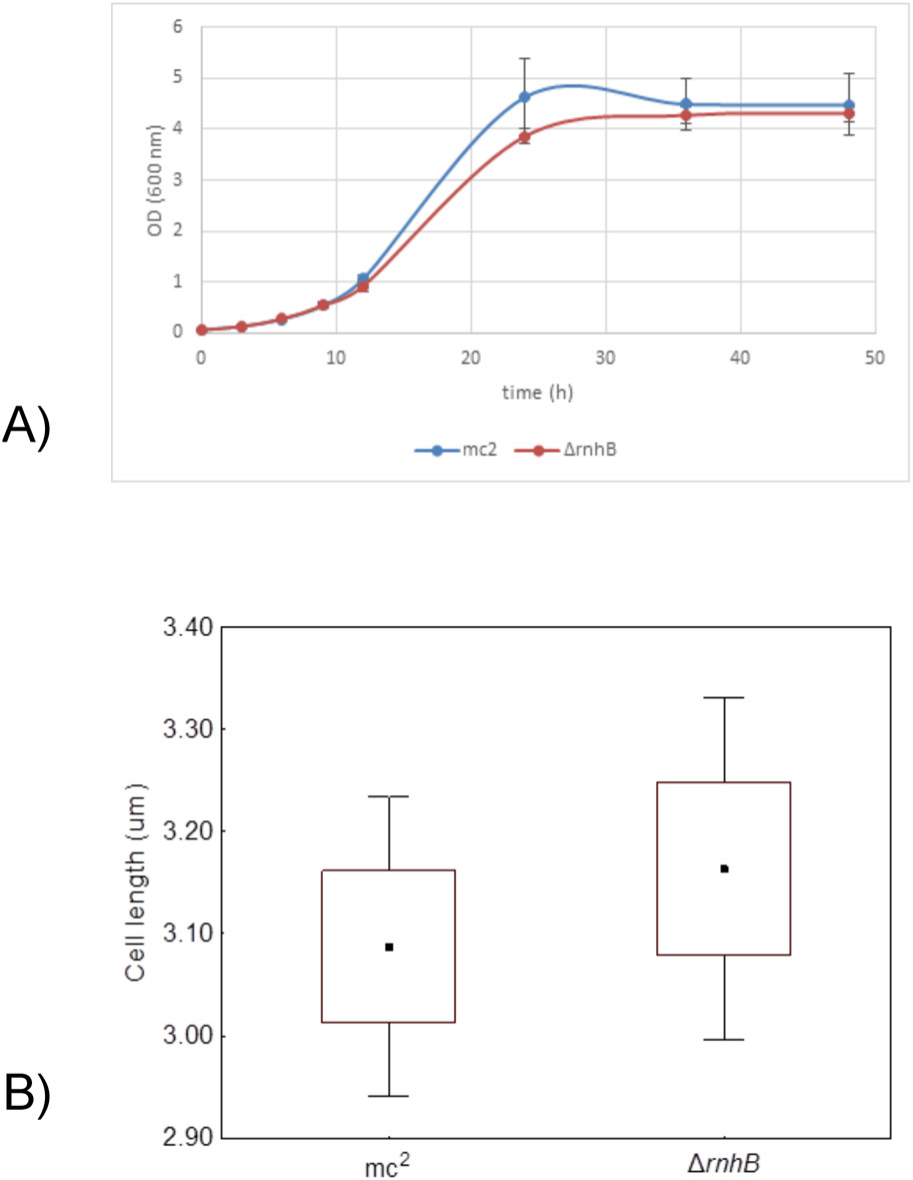
Growth rate and morphology of the ∆*rnhB* mutant and *M. smegmatis* mc^2^ 155 strains. A) Growth rates based on the optical densities of the cultures. B) The cell lengths.

**Growth rate.** We determined whether the deletion of the *rnhB* gene influenced the growth of mutant *M. smegmatis*. To this end, we measured the optical densities of the liquid cultures at determined intervals of time and fitted appropriate growth curves [29]. We did not observe any differences in growth between the ∆*rnhB* and wild-type *M. smegmatis* mc^2^ 155 on 7H9 broth supplemented with OADC when analyzed for asymptotical values (t = 0.83, df = 5, p = 0.44) and growth rate (t = 2.18, df = 5, p = 0.08) (Fig. 4A). The morphology of the mutant cells, in terms of cell length, was not altered (t = 0.68, df = 205, p = 0.50) (Fig. 4B).

**Growth rate in the presence of HU.** Next, we examined the growth rate of mutants lacking *rnhB* in the presence of HU. HU is an inhibitor of class I ribonucleotide reductases, which convert ribonucleoside 5’-diphosphates into deoxyribonucleoside 5’-diphosphates [30]. Therefore, HU is expected to change the balance of NTP pools and increase the amount of rNTPs, while limiting the amount dNTPs. These changes should increase the incorporation of rNTPs into chromosomal DNA. Notably, HU has been shown to influence the DNA content in *M. smegmatis* [31].

**Figure 5:**
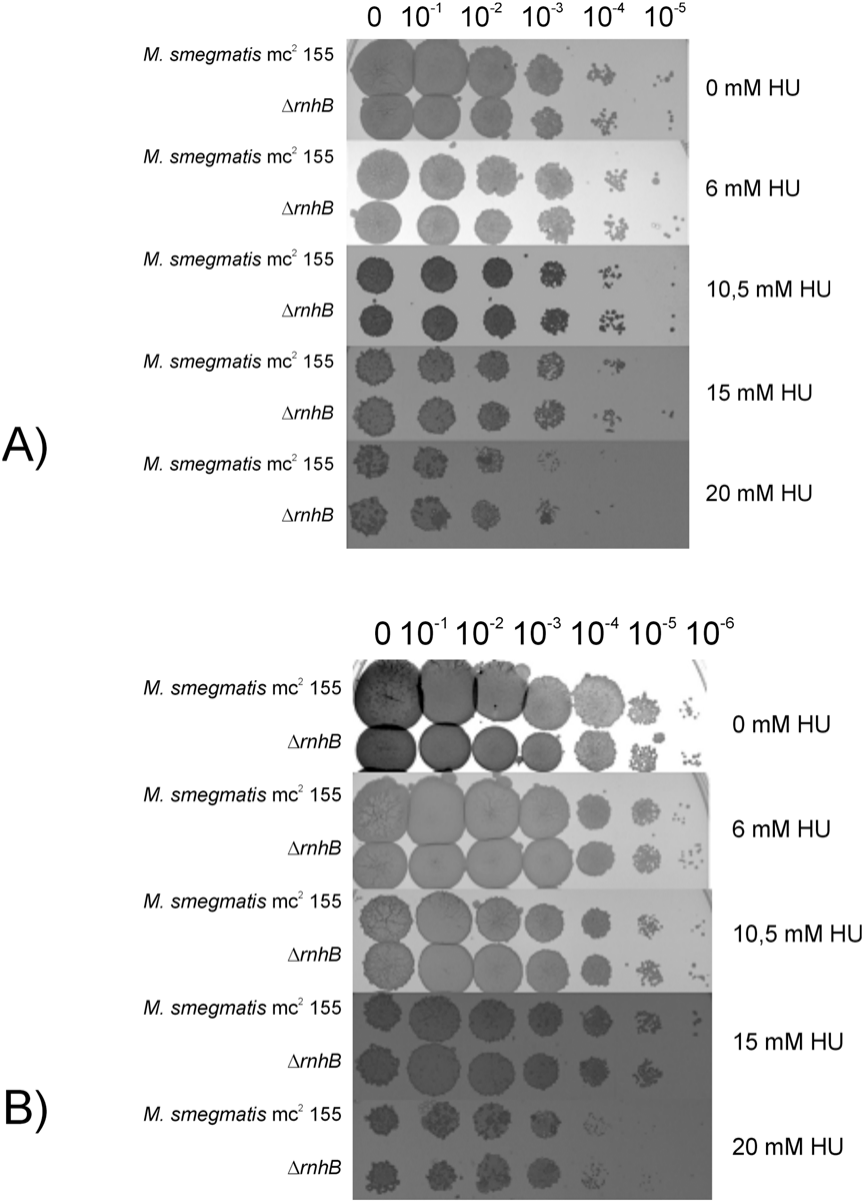
Susceptibility of the ∆*rnhB* mutant and *M. smegmatis* mc^2^ 155 strains to HU. A) Logarithmic and B) stationary phase cultures were serially diluted and plated onto medium containing different concentrations of HU. We observed that 20 mM HU inhibited the growth of the analyzed strains, but we did not observe differences in susceptibility between the strains.

We expected that mutations that alter rNTP removal should affect HU susceptibility levels. Indeed, altered HU susceptibility was observed in RNase H-deficient mutants in yeasts [19, 32]. We did not observe any differences in HU susceptibility in ∆*rnhB* mutants compared with wild type (Fig. 5).

**Figure 6:**
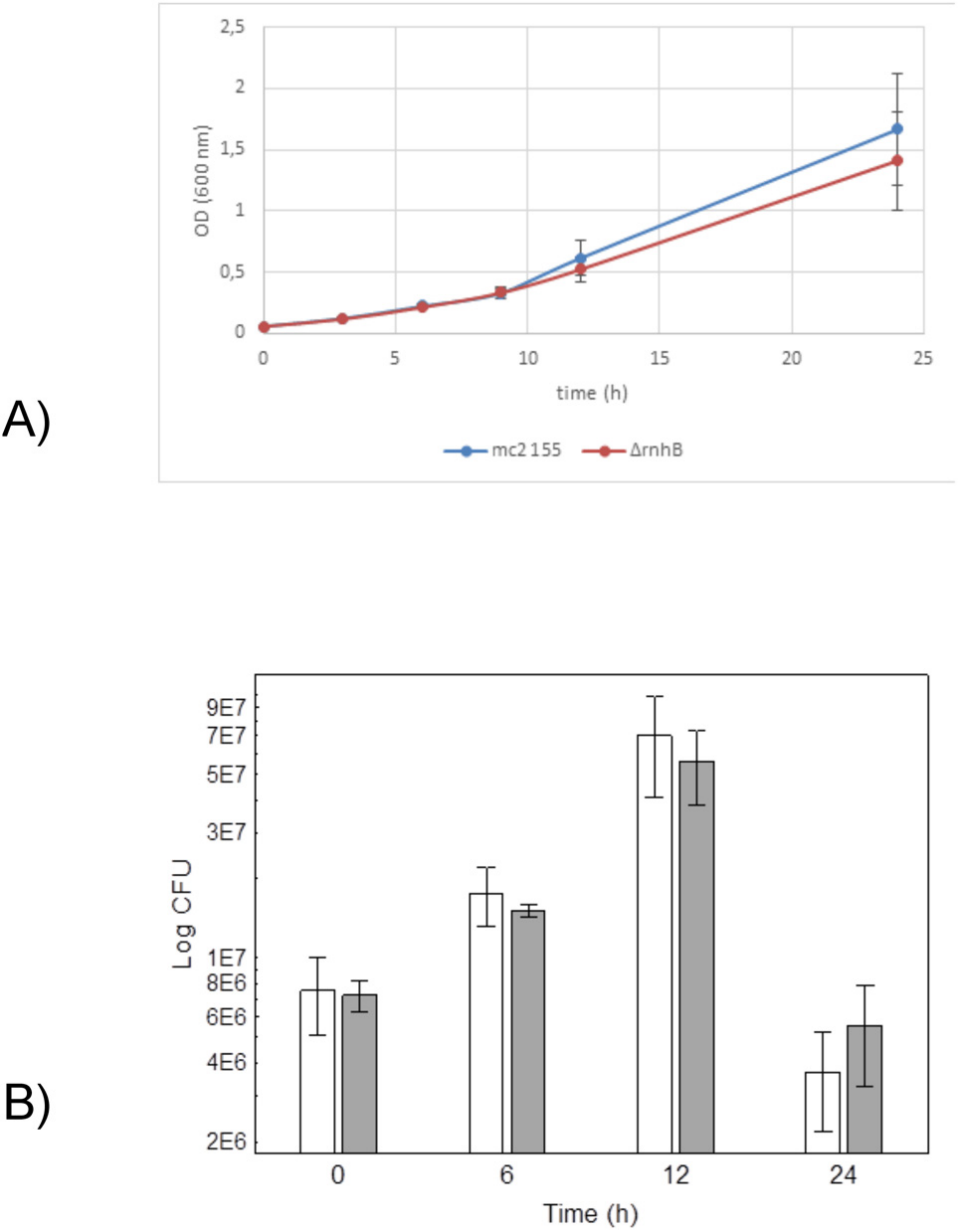
Growth rates in the presence of HU. A) Growth rates based on the optical density of the cultures. B) Growth rates based on the number of colony forming units (white: *M. smegmatis* mc^2^ 155; gray: ∆*rnhB* mutants).

We compared the growth rates of ∆*rnhB* mutants and the wild-type strain in rich 7H9 medium supplemented with HU. The analysis of the growth curves did not reveal any differences in the growth rates of these bacteria in the presence of HU (t = 0.90, df = 8, p = 0.39). These results were consistent with the number of the colony forming units (all p>0.10) (Fig. 6).

**Spontaneous mutation rate.** We also assessed whether the deletion of *rnhB* in *M. smegmatis* influenced the spontaneous mutation rate. We performed the Luria-Delbrück fluctuation test and compared the results obtained from the ∆*rnhA*/∆4305/∆*rnhBattB*::*rnhA*, ∆*rnhA*/∆4305/∆*rnhBattB*::4305 and wild type strains grown in the presence succinate. Briefly, medium inoculated with approximately 100 bacterial cells per ml was divided into 30 parallel cultures and grown for 72 h. Subsequently, 4 cultures of each strain were used to determine the average number of viable cells in the culture, while the remaining cultures were plated onto selective media containing streptomycin. In *M. tuberculosis*, the resistance to streptomycin in approximately 70% of the isolates resulted from mutations in *rpsL* (encoding S12 ribosomal protein), *rrs* (16S rRNA) and *gidB* (ribosome methyltransferase) genes. The cause of the streptomycin resistance in the remaining 30% of the strains remains unknown [33]. While point mutations in *rpsL* and *rrs* caused streptomycin resistance [33], frameshifts and point mutations were responsible for the streptomycin resistance associated with *gidB* [34]. Homologs of *rpsL*, *rss* and *gidB* genes have been identified in *M. smegmatis*.

Determining the number of streptomycin-resistant colonies in each culture facilitated the calculation of the overall mutation rate of each strain. The mutation rate was calculated using FALCOR software (Table 3). We did not observe statistically significant differences in the mutation rate between the analyzed strains (Kruskal-Wallis: H_2, 78_ = 1.64; p = 0.48). Hence, the deletion of *rnhB* did not influence the spontaneous mutation rate in *M. smegmatis*.

**Table 3:** Mutation rates of *M. smegmatis* mc^2^ 155, ∆*rnhA/*∆4305/∆*rnhBattB::rnhA* and ∆*rnhA/*∆4305/∆*rnhBattB::*4305 strains, estimated using the Luria-Delbrück fluctuation test.

**Strain**	**Mutation rate (lower bound-upper bound)**
*M. smegmatis* mc^2^ 155	4.88 (2.8–9.02)
∆*rnhA/*∆4305/∆*rnhBattB::rnhA*	9.03 (3.38–11.85)
∆*rnhA/*∆4305/∆*rnhBattB::*4305	5.6 (3.88–6.75)

### The level of RNase HII substrates in *rnhB*-deficient mutants

**Figure 7:**
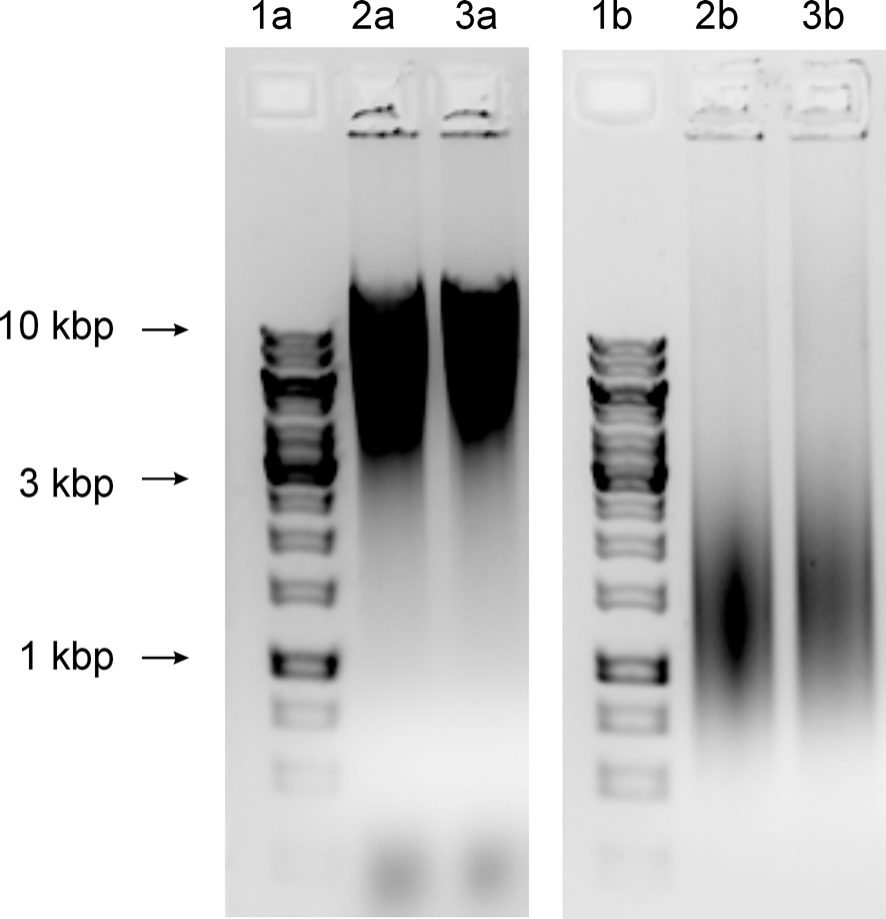
Alkaline hydrolysis of the genomic DNA. DNA was isolated from the ∆*rnhB* mutants and *M. smegmatis* mc^2^ 155. The strains were grown in 7H9 medium supplemented with OADC. The DNA samples were treated with either NaOH or NaCl as a control. The fragmentation of the samples was visualized on alkaline agarose gels. Lanes 1a) GeneRuler 1-kb DNA Ladder, 2a) *M. smegmatis* mc^2^ 155 control DNA, 3a) ∆*rnhB* mutant control DNA, 1b) GeneRuler 1-kb DNA Ladder, 2b) *M. smegmatis* mc^2^ 155 DNA hydrolyzed with NaOH, and 3b) ∆*rnhB* mutant DNA hydrolyzed with NaOH. The level of ribonucleotide incorporated in the DNA of both strains was similar, as we did not observe differences in fragmentation of genomic DNA.

**The level of alkaline degradation of chromosomal DNA.** We compared the level of ribonucleotide incorporation in the DNA isolated from ∆*rnhB* mutant and wild-type strains. The alkaline hydrolysis of genomic DNA has been successfully used in other studies regarding RNase H [35, 36]. As the 3’ phosphodiester bonds of ribonucleotides, but not deoxyribonucleotides, are sensitive to alkali hydrolysis, the fragmentation of genomic DNA under alkaline conditions likely indicates the presence of ribonucleotides, i.e., single ribonucleotides and unresolved RNA primers, embedded within the DNA double helix. Genomic DNA obtained from the mutant and wild-type strains were subjected to alkaline hydrolysis. The control samples were treated with an equal concentration of NaCl. The samples were separated on agarose gels. We expected that an increased level of unprocessed Okazaki primers or an increased level of unremoved single rNTP incorporated within the DNA would show an increase in the degree of genomic DNA fragmentation. We did not observe any differences in the levels of genomic DNA fragmentation under alkaline conditions between the mutant and wild-type strains based on the gel mobility of the alkaline-treated samples (Fig. 7). This observation suggests that neither the unprocessed Okazaki primers nor the single ribonucleotides in the DNA double helix were increased in ∆*rnhB* mutants.

**Figure 8:**
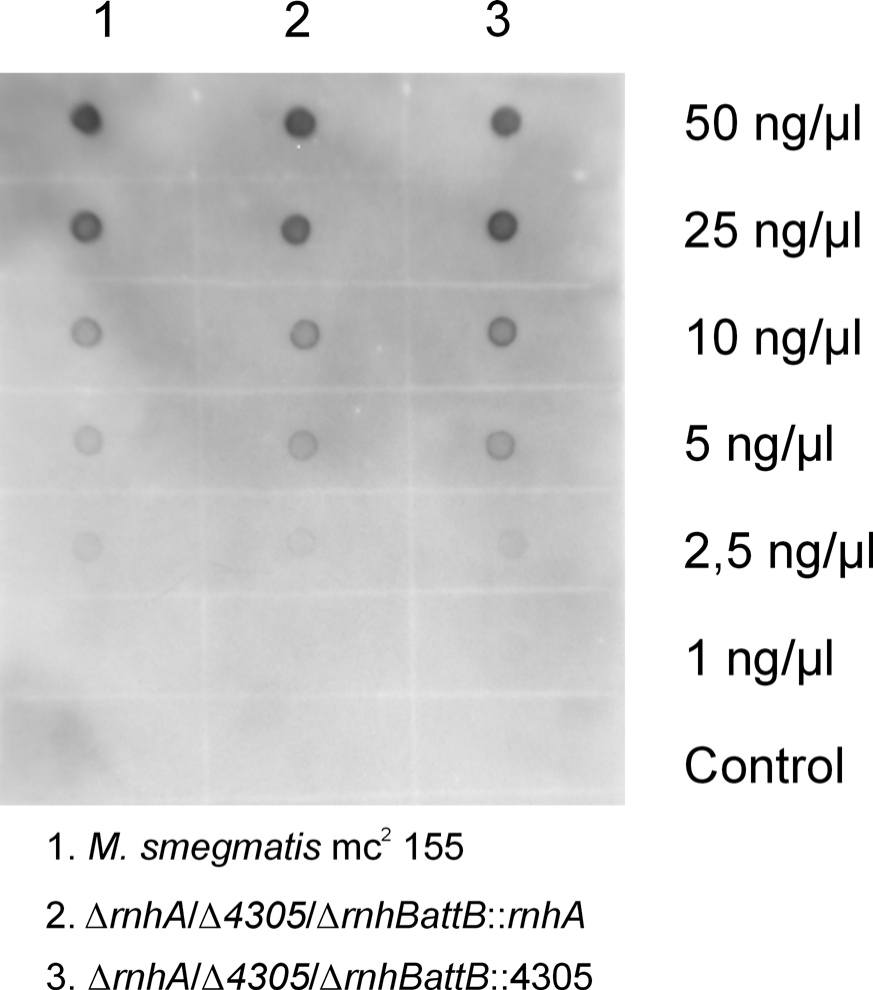
RNA/DNA hybrid level. Comparison of the level of RNA/DNA hybrids in M. smegmatis mc^2^ 155, ∆rnhA/∆4305/∆rnhBattB::rnhA and ∆rnhA/∆4305/∆rnhBattB::4305 strains grown on 7H9 medium in the presence of succinate.

**Immunodetection.** To confirm that the levels of unprocessed Okazaki primers and single ribonucleotides in the DNA double helix were unaltered in *rnhB*-deficient *M. smegmatis*, we performed the immunodetection of RNA/DNA hybrids in nucleic acids isolated from ∆*rnhA*/∆4305/∆*rnhBattB*::*rnhA*, ∆*rnhA*/∆4305/∆*rnhBattB*::4305 and wild-type strains (Fig. 8). These antibodies have previously been used to detect the presence of RNA/DNA hybrids in eukaryotes [37, 38]. We did not observe differences in the strength of the signal generated between different strains, suggesting that the level of RNA/DNA hybrids in these strains was unchanged.

## Discussion

*In silico* analysis revealed an *rnhB* gene encoding a protein with a predicted RNase HII domain. We did not identify any homologs of RNase HIII, consistent with the fact the simultaneous inheritance of RNase HI and RNase HIII in the genome of *M. smegmatis* was avoided due to the functional redundancy of these genes [39].

There has been much confusion regarding the essentiality of RNase HII-encoding genes. Initially, RNase H type II genes were considered essential in *B. subtilis* [40]. However, the Yoshikawa group managed to obtain *B. subtilis* mutants deficient for all RNase H-encoding genes [17]. The growth rate of mutants lacking both RNase HII and RNase HIII was low, suggesting that RNase H proteins are involved in the processing of RNA primers. These suspicions were confirmed through the observation that the overexpression of YpcP exonuclease suppressed the filamentous phenotype and overexpression of the exonuclease domain of PolI in these mutants. RNase HII/RNase HIII mutants showed temperature sensitive growth at 56.5°C [17]. The O’Donell group confirmed the generation of double RNase HII/RNase HIII mutants in *B. subtilis* [35]. RNase HII has been demonstrated as dispensable in *E. coli* [41], although this enzyme was initially considered essential [42].

Using gene replacement through homologous recombination, we generated *M. smegmatis* mutants deficient in *rnhB*, suggesting that this gene is not essential for the survival of *M. smegmatis*. Therefore, either the function of the product of this gene is nonessential for cell survival *in vitro* or there are other genes in the mutant *M. smegmatis* genome whose products have overlapping functions with the mutated gene.

The level of RNase HII substrates and the RNase HII deficiency affect genome stability in both eukaryotes and prokaryotes. In *B. subtilis*, the RNase HII/RNaseHIII/YpcP-deficient mutant displayed a filamentous phenotype, and this phenotype was suppressed through the overexpression of either the deleted genes or the 5’-3’ exonuclease domain of PolI. This phenotype resulted from the induced SOS response, which, in turn, might have resulted from the accumulation of unprocessed Okazaki fragments [17]. The deletion of the 5’-3’ exonuclease domain of PolI in *E. coli*, which is primarily involved in the removal of Okazaki fragments in the absence of DNA damaging agents, increased the mutation rate in terms of frameshift and duplication mutations [43]. It has also been suggested that persisting Okazaki primers destabilize tetranucleotide repeats in *H. influenzae*. This phenotype was associated with the deletion of RNase HI or the Klenow domain of PolI [44].

The deletion of RNase H2 increased the mutation rate in budding yeast [18, 21, 45]. A recent study showed that short, 2–5 bp deletions observed in budding yeast mutants defective for RNase H2 result from topoisomerase I activity, and the deletion of topoisomerase I in RNase H2 mutants restored the mutation rate associated with these changes in the wild type [21]. Notably, the rates for mutations other than 2–5 bp deletions were not restored to the wild type in double the RNase H2/topoisomerase I mutant [21].

It has been suggested that ribonucleotide incorporated in DNA signals a newly synthesized strand for MMR. In *E. coli*, the MMR MutH enzyme recognizes and cleaves a newly synthesized strand at hemimethylated GATC sites [46]. *E. coli* mutants deficient in RNase HII grow as fast as wild type and show no increase in spontaneous mutation rates. Therefore, the authors concluded that ribonucleotides persisting in the *E. coli* chromosome are non mutagenic [35]. However, contrasting observations were shown in *B. subtilis*, which lacks a homolog of MutH. Instead MutL exhibits endonuclease activity that is not present in *E. coli*. It has been proposed that MutL requires a signal to direct this enzyme to the newly synthesized DNA strand. Signals from Okazaki fragments might be functional for the lagging strand, but not for the leading strand. Therefore, it has been proposed that these signals are provided from the ribonucleotides incorporated within the newly synthesized strand. To examine this hypothesis, the authors measured the spontaneous mutations rates in RNase H type II-deficient *B. subtilis* mutants. The deletion of RNase HII resulted in 2.4-fold increase in mutation rates, while the deletion of RNase HIII only increased mutation rates 1.3-times compared with wild type. Double mutation resulted in a five-fold increase in the mutation rates. These authors speculated that the increased mutation rate corresponded to the 10% decline in MMR efficiency [35]. The hypothesis that ribonucleotides embedded within DNA act as a strand discrimination factor during MMR has been confirmed in eukaryotes [47].

It has been shown that yeast DNA polymerase ε bypasses a single rNTP present within the DNA template [18], and the presence of ribonucleotides in the template delays bacterial replisome progression 4–30-fold [35]. Notably, mouse embryos deficient in RNase H2 show arrested development and display an increased number of ribonucleotides in the genomic DNA [48]. Thus, ribonucleotides embedded within DNA duplex might constitute a barrier for replication fork progression. While this barrier is impossible to circumvent in higher eukaryotes, based on the essentiality of RNase H, in yeast, the double deletion of RNase H1 and RNase H2 sensitizes the cells to replication stress-inducing agents, such as HU and methyl methanesulfonate [19]; however, increased HU susceptibility after single RNase H2 deletion has been observed [32]. Additionally, RNase H deletion induces the constant activation of post-replication repair, although the mechanisms of this phenomenon are poorly understood [19].

Primary phenotypic analysis of the growth rate and cell morphology showed that ∆*rnhB M. smegmatis* mutants exhibit growth similar to the wild-type strain, suggesting that ribonucleotides incorporated within DNA double helix after *rnhB* deletion do not constitute a barrier for replication fork progression. The growth rate of ∆*rnhB* mutants remained unaltered, even in the presence of HU, which is considered to increase ribonucleotide incorporation. These observations are in contrast to the data obtained in *B. subtilis* [17], yeast [19] and higher eukaryotes [36]. Moreover, Luria-Delbrück fluctuation analyses did not reveal increased mutation rates in RNase HII-deficient mutants. However, *M. smegmatis* does not possess homologs of the MMR system, therefore an MMR defect cannot be expected. Indeed, when we analyzed the level of RNase HII substrates in *rnhB*-deficient cells, based on the levels of alkaline degradation of the chromosomal DNA or the immunodetection of RNA/DNA hybrids, we did not observe differences between mutant and wild-type strains. Therefore, in contrast to *B. subtilis* [35], yeast [18] and higher eukaryotes [36], the RNase HII deletion did not increase the level of RNase HII substrates in *M. smegmatis*.

Thus, we concluded that the RNase HII activity in *M. smegmatis* cells after ∆*rnhB* deletion is sufficient to remove RNA/DNA hybrids to wild-type levels, and the genome stability in these deletion mutants is unaffected. Therefore, proteins other than RnhB proteins must be involved in the removal of RNase HII substrates in *M. smegmatis*. Based on the data obtained from previous studies, these proteins might include PolI [17], MSMEG5849 [24] and/or RNase HI [19]. For example in *E. coli* the main enzyme responsible for the removal of RNA primers during Okazaki fragment maturation is thought to be polymerase I (PolI) encoded by *polA* [14, 15, 16]. This enzyme possesses a number of enzymatic activities: 5’-3’ DNA dependent DNA polymerase [49], 5’-3’ RNA dependent DNA polymerase [50], 3’-5’ exonuclease activity [51] and 5’-3’ exonuclease activity [51]. There seems to be a lot of confusion regarding essentiality of PolI in bacteria. While polymerase domain of PolI can be inactivated in both *E. coli* [50] and *M. smegmatis* [52], it seemed that 5’-3’ exonuclease activity is essential for survival of *E. coli* [53]. In fact, it has been speculated that PolI temperature sensitive mutant strains are lethal precisely due to the failures in removal of RNA primers during DNA synthesis [54]. Other authors argued that *polA* mutant is in fact viable on minimal medium, but not on rich medium [55]. The same report stated that complementation of the mutant strain with either 5’-3’ exonuclease portion of PolI or polymerase 3’-5’ exonuclease portions restores the viability of the mutant on rich medium [55]. Another group was able to obtain a normally growing *polA* mutant on LB medium [43]. Finally, Yoshikawa group showed that *polA* can be deleted from the genome of *B. subtilis*, however only in the presence of other gene, *ypcP*, providing 5’-3’ exonuclease activity. They observed similar phenomenon in *E. coli*, when 5’-3’ activity is provided by *ypcP* homolog *xni*. The *polA* mutant that they obtained presented temperature sensitivity and did not grow at 56.5°C [17].

It was shown in *E. coli*, that RNase HI might also be involved in RNA primers removal, however its role is thought to be restricted to cleavage of longer RNA fragments [14]. Again there are contradictory reports regarding its essentiality [56, 57, 58]. The authors claiming to have obtained RNase HI deficient mutants stated that the mutants are rich broth sensitive [57, 58], which could explain the failure in obtainment of the mutant by Kanaya and Crouch.

Additional factor presumably capable of removing RNase HII substrates is a protein encoded by MSMEG5849. This protein might be sufficient to remove both single ribonucleotides embedded within DNA double helix and Okazaki primers. MSMEG5849 possesses two enzymatic activities- an RNase HII and RelA-SpoT nucleotydyl transferase domain responsible for ppGpp synthesis [24]. Until 2012 the RNase HII domain of this protein was described as a domain of unknown function 429.
